# Dietary Resveratrol Butyrate Monoester Supplement Improves Hypertension and Kidney Dysfunction in a Young Rat Chronic Kidney Disease Model

**DOI:** 10.3390/nu15030635

**Published:** 2023-01-26

**Authors:** You-Lin Tain, Chi-I Chang, Chih-Yao Hou, Guo-Ping Chang-Chien, Sufan Lin, Chien-Ning Hsu

**Affiliations:** 1 Department of Pediatrics, Kaohsiung Chang Gung Memorial Hospital, Kaohsiung 833, Taiwan; 2 College of Medicine, Chang Gung University, Taoyuan 330, Taiwan; 3 Department of Biological Science and Technology, National Pingtung University of Science and Technology, Pingtung 912, Taiwan; 4 Department of Seafood Science, National Kaohsiung University of Science and Technology, Kaohsiung 811, Taiwan; 5 Institute of Environmental Toxin and Emerging-Contaminant, Cheng Shiu University, Kaohsiung 833, Taiwan; 6 Super Micro Mass Research and Technology Center, Cheng Shiu University, Kaohsiung 833, Taiwan; 7 Center for Environmental Toxin and Emerging-Contaminant Research, Cheng Shiu University, Kaohsiung 833, Taiwan; 8 Department of Pharmacy, Kaohsiung Chang Gung Memorial Hospital, Kaohsiung 833, Taiwan; 9 School of Pharmacy, Kaohsiung Medical University, Kaohsiung 807, Taiwan

**Keywords:** gut microbiota, oxidative stress, resveratrol, chronic kidney disease, nitric oxide, hypertension, renin-angiotensin system

## Abstract

Chronic kidney disease (CKD) remains a public health problem. Certain dietary supplements can assist in the prevention of CKD progression. In this regard, resveratrol is a polyphenol and has a potential therapeutic role in alleviating CKD. We previously utilized butyrate in order to improve the bioavailability of resveratrol via esterification and generated a resveratrol butyrate monoester (RBM). In this study, the hypothesis that RBM supplementation is able to protect against kidney dysfunction and hypertension was tested by using an adenine-induced CKD model. For this purpose, three-week-old male Sprague Dawley rats (n = 40) were equally categorized into: group 1—CN (sham control); group 2—CKD (adenine-fed rats); group 3—REV (CKD rats treated with 50 mg/L resveratrol); group 4—MEL (CKD rats treated with 25 mg/L RBM); and group 5—MEH (CKD rats treated with 50 mg/L RBM). At the end of a 12-week period, the rats were then euthanized. The adenine-fed rats displayed hypertension and kidney dysfunction, which were attenuated by dietary supplementation with RBM. The CKD-induced hypertension coincided with: decreased nitric oxide (NO) bioavailability; augmented renal protein expression of a (pro)renin receptor and angiotensin II type 1 receptor; and increased oxidative stress damage. Additionally, RBM and resveratrol supplementation shaped distinct gut microbiota profiles in the adenine-treated CKD rats. The positive effect of high-dose RBM was shown together with an increased abundance of the genera *Duncaniella*, *Ligilactobacillus*, and *Monoglobus*, as well as a decrease in *Eubacterium* and *Schaedierella*. Importantly, the mechanism of action of the RBM supplementation may be related to the restoration of NO, rebalancing of the RAS, a reduction in oxidative stress, and alterations to the gut microbiota. Moreover, RBM supplementation shows promise for the purposes of improving CKD outcomes and hypertension. As such, further translation to human studies is warranted.

## 1. Introduction

The high prevalence of chronic kidney disease (CKD) is a major contributor to the global burden of disease [1]. Despite the advances made in CKD management, the global prevalence of the disease has continued to rise. As adults who have CKD can contract the disease in early life [2,3], the early detection within childhood and the treatment of children who have CKD can be effective in alleviating the global burden of CKD [4]. Several animal models of CKD have been established in order to investigate new therapeutic approaches for the prevention of CKD progression. This has been performed in conjunction with the addition of adenine to the diets of rats, which has been found to be an animal model that mimics human CKD [5]. Our prior work indicates that adenine-treated young rats developed major characteristics of pediatric CKD, including kidney function impairment, hypertension, renal hypertrophy, and increases in uremic toxins [6].

Growing evidence suggests that a plant-based diet may exert beneficial effects with respect to treating CKD and its associated complications [7,8]. Resveratrol, a polyphenol found in fruits and vegetables, possesses potential health benefits and therapeutic properties that could be effective in terms of combating kidney disease [9]. Resveratrol is known to possess wide ranges of renoprotective effects, such as: anti-inflammation and antioxidant properties; restorative of NO bioavailability; prebiotic effects; and a capacity for rebalancing the renin–angiotensin system (RAS), etc. [10,11]. However, the low bioavailability of resveratrol diminishes its efficacy and limits its clinical utility [12].

We previously utilized short-chain fatty acids (SCFAs) in order to improve the biological activity and bioavailability of resveratrol [13,14]. Our prior work demonstrated that the esterification of resveratrol with butyrate enabled one to generate a mixture of resveratrol, one resveratrol diester, and two resveratrol monoesters [13,14]. Additionally, our data demonstrated that the mixtures of resveratrol butyrate esters (RBEs) have a higher antioxidant capacity than resveratrol itself [14]. While certain dietary interventions have demonstrated a benefit in slowing CKD progression [7,8]—as well as being shown to modify the gut microbiota—the direct link from the perspective of targeting gut microbiota in order to prevent and control CKD has only recently come under investigation and remains, as of yet, incompletely understood [15].

Although our previous study demonstrated that RBEs effectively attenuate CKD-induced oxidative damage and display a BP-lowering effect [6], the question regarding what beneficial effect is related to which component of the RBEs remains unclear. Recently, we purified a resveratrol butyrate monoester (RBM), as well as a 3-O-butyrylresveratrol, from the RBE mixtures [13]. In this study, we examined the effectiveness of RBM at low and high doses versus that of resveratrol in the treatment of CKD. In addition, we also explored the protective mechanisms by using an adenine-induced CKD young rat model.

## 2. Materials and Methods

### 2.1. Synthesis of Resveratrol Butyrate Esters 

The mixtures of RBEs were synthesized based on our protocol, as described previously [13]. The butyric acid (ACROS, Morris Plains, NJ, USA) was mixed with resveratrol (TCI Development Co., Ltd., Shanghai, China) in tetrahydrofuran (Echo Chemical Co., Ltd., Miaoli County, Taiwan). Later, N-ethyl-N′-(3-dimethylaminopropyl) carbodiimide (Sigma-Aldrich, Saint Louis, MO, USA) and 4-dimethylaminopyridine (Sigma-Aldrich, Saint Louis, MO, USA) were added. Then, the esterification reaction was conducted by avoiding light for 48 h. Upon completion, the reaction mixture was mixed with distilled water and filtered in order to obtain the precipitated RBEs. The RBEs were kept within a −20 °C freezer.

### 2.2. Purification of Resveratrol Monoester

The resveratrol monoester was purified and prepared according to our previous protocol [13]. Briefly, the reaction mixture (24 g) was subjected to chromatography based on silica gel column elution with a gradient of increasing polarity of CH_2_Cl_2_–acetone, followed by methanol, in order to provide 126 fractions. These fractions were combined in order to form Fractions 1–17 based on thin-layer chromatography results. Fraction 11 (1.9 g) was purified using silica gel column elution with CH_2_Cl_2_–acetone (9:1) and further eluted with CH_2_Cl_2_–EtOAc (80:1–4:1) in order to provide 12 fractions (11A–11L). Later, we purified Fraction 11F by using crystallization with acetone to acquire 3-O-butyrylresveratrol (856 mg). Following purification of the mixture of RBEs, we identified the structure type of 3-O-butyrylresveratrol as an RBM and used it for the following experiments [13].

### 2.3. Animal Study

All experimental procedures were carried out at Kaohsiung Chang Gung Memorial Hospital with prior approval by the Institutional Animal Care and Use Committee (approval number, 2021081204). The Sprague Dawley (SD) rats were purchased from BioLASCO Taiwan Co., Ltd. (New Taipei City, Taiwan) and housed in our AAALAC-accredited animal facility. We only studied the male rats; this was performed due to the fact that females are less affected by hypertension at a younger age [16]. At 3 weeks, rats were fed regular (CN group; N = 8) or 0.25% adenine chow (CKD group; N = 32) for 3 weeks. One group of adenine-fed CKD rats received resveratrol (50 mg/L, REV group) in their drinking water for 6 weeks (weeks 6–12). The other CKD rats were treated with a low dose (25 mg/L in drinking water, MEL group) or a high dose (50 mg/L in drinking water, MEH group) of resveratrol monoester for 6 weeks (weeks 6–12).

We used an indirect tail-cuff method (CODA, Kent Scientific Corp., Torrington, CT, USA) to determine BP [6]. Before measurement, rats were acclimated to a restraint box and tail-cuff inflation in a quiet area for one week. At 12 weeks of age, the rats were then euthanized. Fresh stool samples were gathered and kept at −20 °C until required for analysis. The blood samples were collected into heparinized tubes. We harvested the kidneys after perfusion with phosphate-buffered saline and stored them within a −80 °C freezer. Finally, we determined the plasma creatinine concentration by using high-performance liquid chromatography (HPLC, HP series 1100; Agilent Technologies Inc., Santa Clara, CA, USA).

### 2.4. Analysis of NO Parameters

L-arginine is the substrate for NO synthase (NOS) used to generate NO, while NOS can be inhibited by symmetric and asymmetric dimethylarginine (SDMA and ADMA) [17]. Accordingly, the ratio of L-arginine to ADMA has been applied as an index of NO bioavailability [18]. Therefore, we determined the plasma levels of L-arginine, ADMA, and SDMA in order to elucidate the impact of CKD and resveratrol monoester on the NO-signaling pathway by utilizing the HPLC method (Agilent Technologies Inc.) with o-phthalaldehyde (OPA)/3-mercaptopropionic acid (3-MPA) fluorescent derivatives [7].

### 2.5. Western Blotting

The protocol for determining the protein abundance of NOS and RAS components via Western blot analysis was based as per our previous reports [19]. Equal amounts (200 μg) of kidney cortical protein extracts were loaded. After the separation of proteins via SDS-PAGE, the proteins were then transferred onto nitrocellulose membranes. For this, we used Ponceau S red (PonS) as the loading controls. Following staining with the PonS stain solution (0.2% *w*/*v* in 1% acetic acid), the membranes were imaged and saved in the TIFF file for the purposes of quantitative analysis. After washing the membranes, the nonspecific sites were blocked through incubation in the blocking buffer (5% milk in 0.05% TBS-T). Later, the membranes were incubated with the indicated primary antibodies, as listed in Table 1. Following the incubation loaded with a secondary antibody, the immuno-reactive bands were pictured using enhanced chemiluminescence (PerkinElmer, Waltham, MA, USA) and digitized by Bio-Rad Quantity One (Bio-Rad, Hercules, CA, USA). In this regard, the protein abundance was given as the integrated optical density (IOD)/PonS.

### 2.6. Detection of Oxidative Stress via 8-OHdG Immunostaining

It must be noted that 8-hydroxydeoxyguanosine (8-OHdG) is considered a biomarker of oxidative DNA damage [20]. The kidney sections were deparaffinized and rehydrated through xylene and a decreasing alcohol gradient. Furthermore, we blocked the kidney sections with immunoblock (BIOTnA Biotech., Kaohsiung, Taiwan), followed by incubation periods of 2 h with an anti-8-OHdG antibody (1:100, JaICA, Shizuoka, Japan). The immunohistochemistry was performed using the polymer-horseradish peroxidase (HRP) Detection System with 3,3′-diaminobenzidine (DAB) (BIOTnA Biotech). Moreover, the scoring of 8-OHdG-stained cells in high-power fields (200×) within the kidney sections was performed by counting the numbers from the kidney sections.

### 2.7. 16S Metagenomic Sequencing

Next, we extracted microbial DNA from the fecal samples. The bacterial 16S rRNA gene was used for the purposes of metagenomics analysis at Biotools Co., Ltd. (New Taipei City, Taiwan) [6]. The full-length 16S genes were amplified using barcode primers that were adapted for SMRTbell library preparation and sequencing (PacBio, Menlo Park, CA, USA). All downstream analyses of these sequences were conducted using the QIIME2 software package [21]. In addition, a phylogenetic tree was generated from the amplicon sequence variants (ASVs) via FastTree (QIIME2). The α-diversity indices, Shannon index, and Faith’s phylogenetic diversity (PD) index, were all determined at the ASV level. We used two β-diversity indices to characterize the similarities between communities across groups: the principal coordinate analysis (PCoA) of unweighted UniFrac distance and the analysis of similarities (ANOSIM).

### 2.8. Statistical Analysis 

Data were presented as the mean ± the standard error of the mean (SEM). The data were then subjected to a one-way analysis of variance (ANOVA), followed by Tukey’s post hoc test. A *p*-value <0.05 was tested, as it was considered to indicate statistical significance, by using the Statistical Package for the Social Sciences software (SPSS Inc., Chicago, IL, USA).

## 3. Results

### 3.1. Weight, BP, and Renal Function

The mortality rate was 12.5% in each group, except for in the CN group (which was zero (Table 2)). The body weight (BW) and kidney weight (KW) in the CN group were lower than those in the other groups. The REV group exhibited a higher KW-to-BW ratio when compared to that in the CN group. Additionally, the adenine diet-induced increases in systolic BP and mean arterial pressure were mitigated in the REV, MEL, and MEH groups. Furthermore, it was found that only the high-dose resveratrol monoester restored diastolic BP back to a normal level (Table 2). Moreover, the plasma creatinine level was increased in the CKD group, which was found to be prevented by resveratrol or resveratrol monoester treatment. As can be seen from our results, the adenine diet-induced group demonstrated body weight gains, as well as hypertension and kidney function impairment (~30% reduction). Furthermore, the adenine diet-induced hypertension and kidney function impairment were improved by either resveratrol or resveratrol monoester treatment. However, this phenomenon engendered a negligible effect with respect to mortality and body weight.

### 3.2. Oxidative Stress Damage

**Figure 1:**
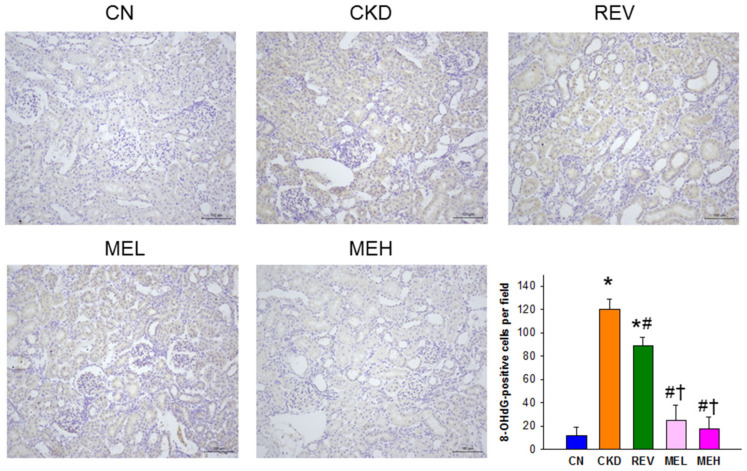
Light micrographs illustrating immunostaining for 8-OHdG in the rat kidneys (200×). n = 8/group; * *p* < 0.05 vs. CN; # *p* < 0.05 vs. CKD; and † *p* < 0.05 vs. REV.

The beneficial effects of resveratrol with respect to kidney disease and hypertension have been linked to its antioxidant properties [10]. We therefore examined whether resveratrol or resveratrol monoester protects against CKD-induced oxidative stress by determining the renal 8-OHdG expression. There was intense-to-moderate staining of 8-OHdG in the glomeruli and tubules in the CKD and REV groups. However, weak staining was found in the CN, MEL, and MEH groups instead (Figure 1). It must be noted that there was no significant difference in the staining pattern between the MEL and MEH groups.

### 3.3. Nitric Oxide Pathway

**Figure 2:**
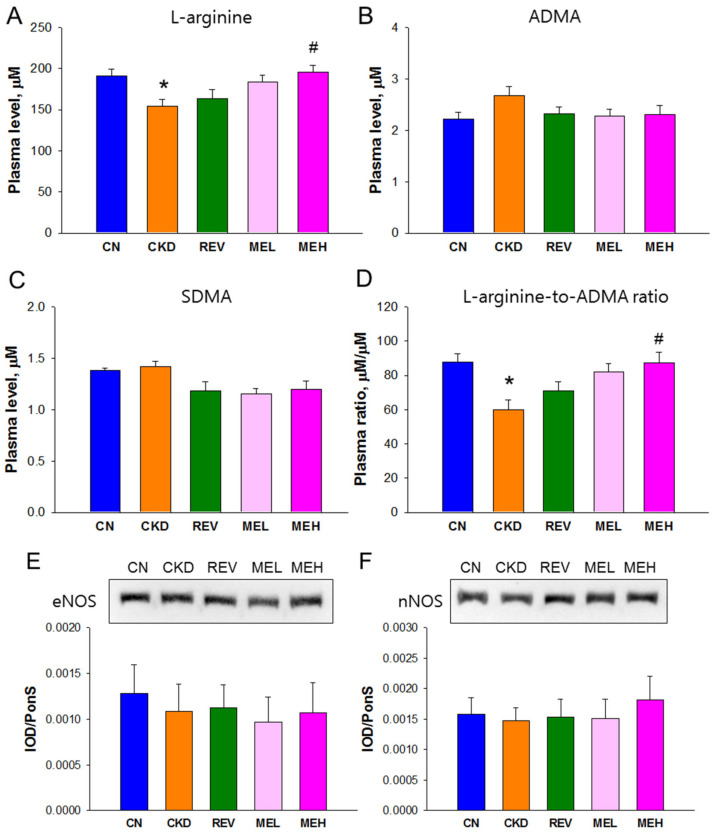
NO parameters and nitric oxide synthase (NOS) expression in rats at 12 weeks of age. Plasma concentrations (**A**) L-arginine, (**B**) asymmetric dimethylarginine (ADMA), (**C**) symmetric dimethylarginine (SDMA), and (**D**) the L-arginine-to-ADMA ratio. Protein expression of (**E**) endothelial NOS (eNOS, 140 kDa) and (**F**) neuronal NOS (nNOS, 155 kDa) in rat kidneys. n = 8/group; * *p* < 0.05 vs. CN; and # *p* < 0.05 vs. CKD.

We next analyzed the NO pathway (Figure 2). This was performed due to the fact that the beneficial effect of resveratrol has been linked to the restoration of NO [10]. We found that the adenine diet-induced group showed decreases in plasma L-arginine concentrations (Figure 2A), as well as in the ratio of L-arginine to ADMA (Figure 2D) in the CKD group, which was also decreased in comparison with that in the CN group. These changes were restored by high-dose resveratrol monoester treatment. However, the plasma concentrations of ADMA and SDMA did not differ among the five groups (Figure 2B,C). We also evaluated the protein abundance of eNOS and nNOS via the Western blot method (Figure 2E,F). Among the five groups, there was no difference in the expression of both NOSs.

### 3.4. Renin-Angiotensin System

**Figure 3:**
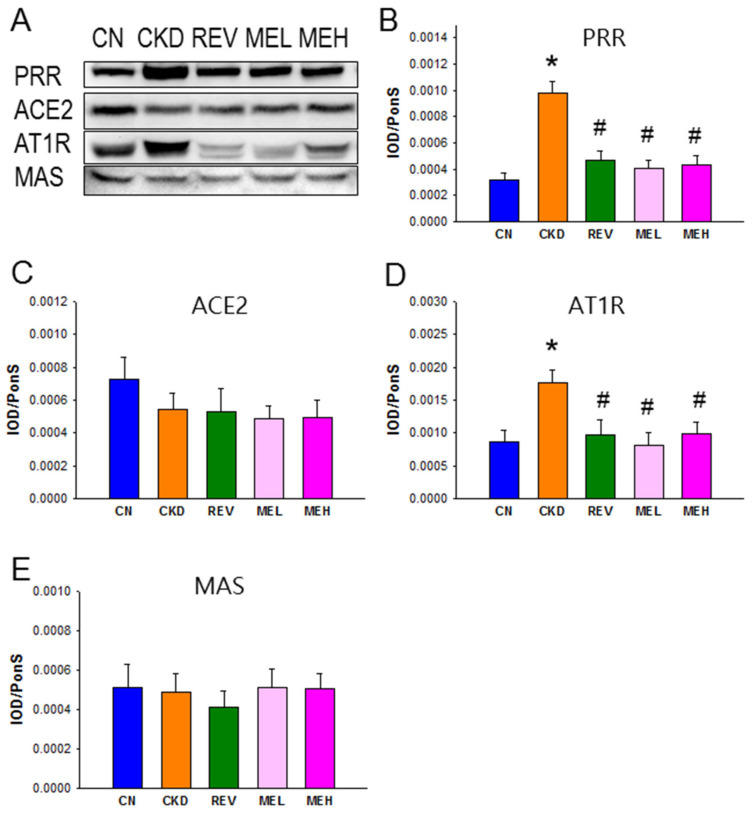
Protein expression of the renin–angiotensin (RAS) system components in rats at 12 weeks of age. (**A**) Representative Western blots showed (pro)renin receptor (PRR, 39 kDa), angiotensin-converting enzyme 2 (ACE2, 90 kDa), angiotensin II type 1 receptor (AT1R, 43 kDa), and angiotensin (1–7) receptor MAS (37 kDa) bands. Relative abundance of renal cortical (**B**) PRR, (**C**) ACE2, (**D**) AT1R, and (**E**) MAS, as quantified. n = 8/group; * *p* < 0.05 vs. CN; and # *p* < 0.05 vs. CKD.

We next evaluated renal expression of the RAS components (Figure 3), due to the fact that the RAS has a pathological role in hypertension. The protein expression of PRR and AT1R, i.e., the two RAS components that favor vasoconstriction, were higher in the CKD group than in the other groups (Figure 3A,D). In addition, ACE2 and MAS—i.e., the two proteins belonging to the non-classical RAS axis—showed no significant differences among the five groups.

### 3.5. Offspring Metagenome

**Figure 4:**
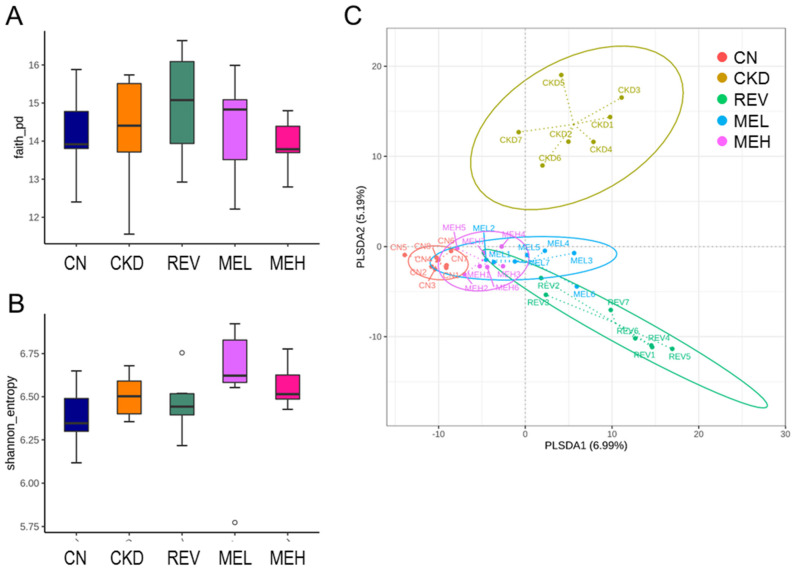
Gut microbiota communities in five experimental groups. (**A**) Faith’s phylogenetic diversity (PD) index and (**B**) the Shannon index were applied to analyze α-diversity. (**C**) β-Diversity analysis was assessed via partial least squares discriminant analysis (PLSDA) in five clusters, whereby each dot represents the microbiota of a single sample, and the color of the dot indicates the group and the sample. Each axis percentage reflects how much variation was accounted for by the 1-dimension.

The compositions of bacterial communities were evaluated by assessing two major ecological parameters: the Faith’s PD index (a metric for phylogenetic richness, see Figure 4A) and the Shannon index (a metric for combined richness and evenness, see Figure 4B). Microbial richness and evenness were found to show no differences between each group. In order to visualize whether the five groups were significantly different in terms of their microbial communities (i.e., β-diversity), 2-dimensional scatterplots were generated based on a PLSDA metric (Figure 4C). A clear separation was observed in the PLSDA between most clusters, with the only exceptions being found between the MEL and MEH groups. Similarly, the ANOSIM test showed statistically significant differences between most groups (all groups recorded at *p* < 0.05), apart from the difference between the MEL and MEH groups, which did not reach the significance threshold (*p* = 0.097).

**Figure 5:**
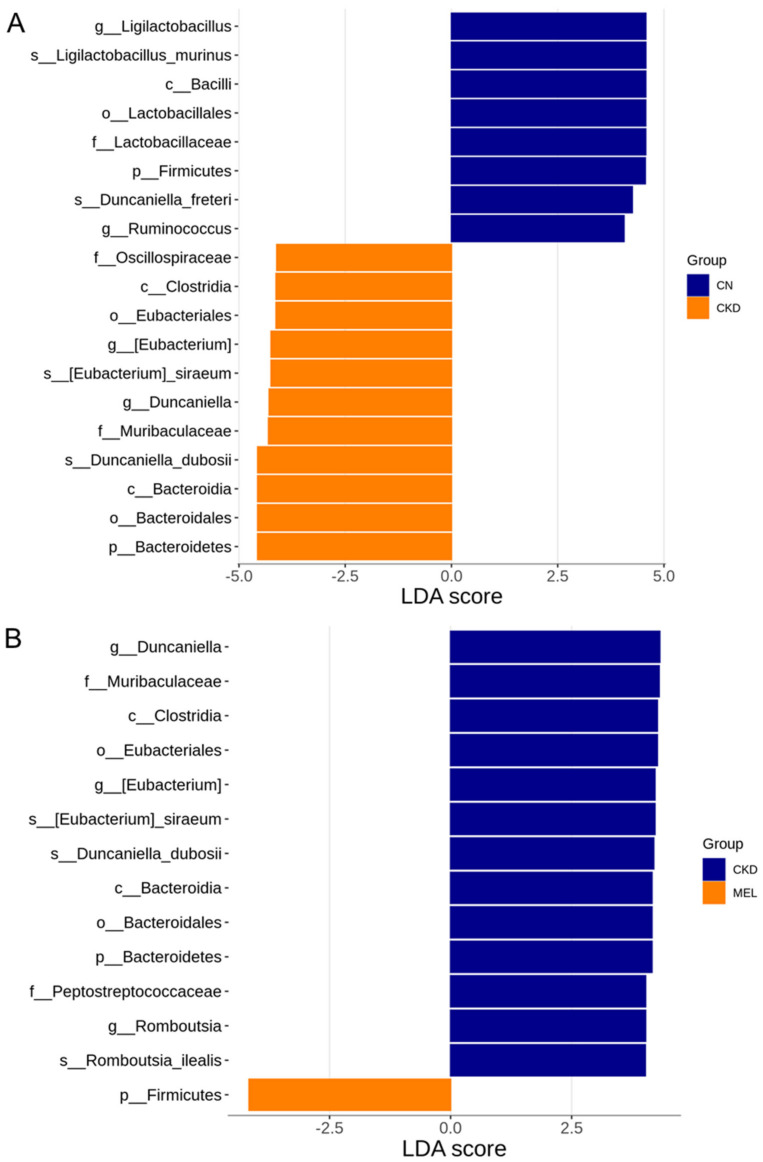

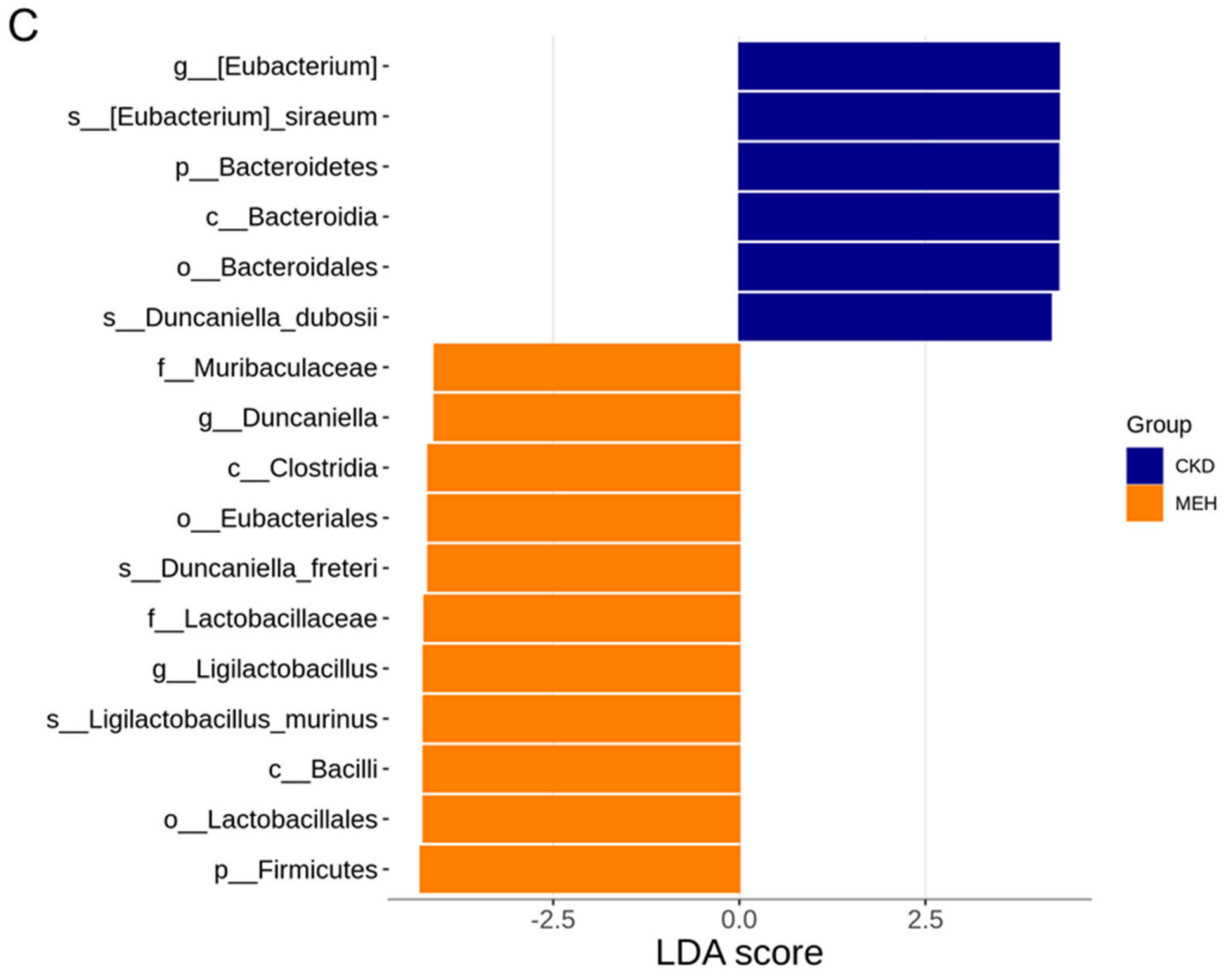
Linear discriminant analysis (LDA) along with effect-size measurements applied to present the most enriched and depleted taxa in: (**A**) the CN (blue) and CKD (orange) groups; (**B**) the CKD (blue) and MEL (orange) groups; and (**C**) the CKD (blue) and MEH (orange) groups. The LDA score threshold was set to greater than 4.

A total of 19 taxa significantly differed in relative abundances in the comparison between the CN and CKD groups (Figure 5A). Specifically, the LEfSe was performed at the genus level, which indicated that *Ligilactobacillus* and *Ruminococcus* were highly enriched in the CN group, while *Eubacterium* and *Duncaniella* were found in lower quantities. Figure 5B reveals that three genera—*Duncaniella*, *Eubacterium* and *Romboutsia*—were more abundant in the CKD group than in the MEL group. High-dose RBE treatment resulted in higher quantities of the genera *Duncaniella* and *Ligilactobacillus*, but a lesser abundance of *Eubacterium* (Figure 5C).

**Figure 6:**
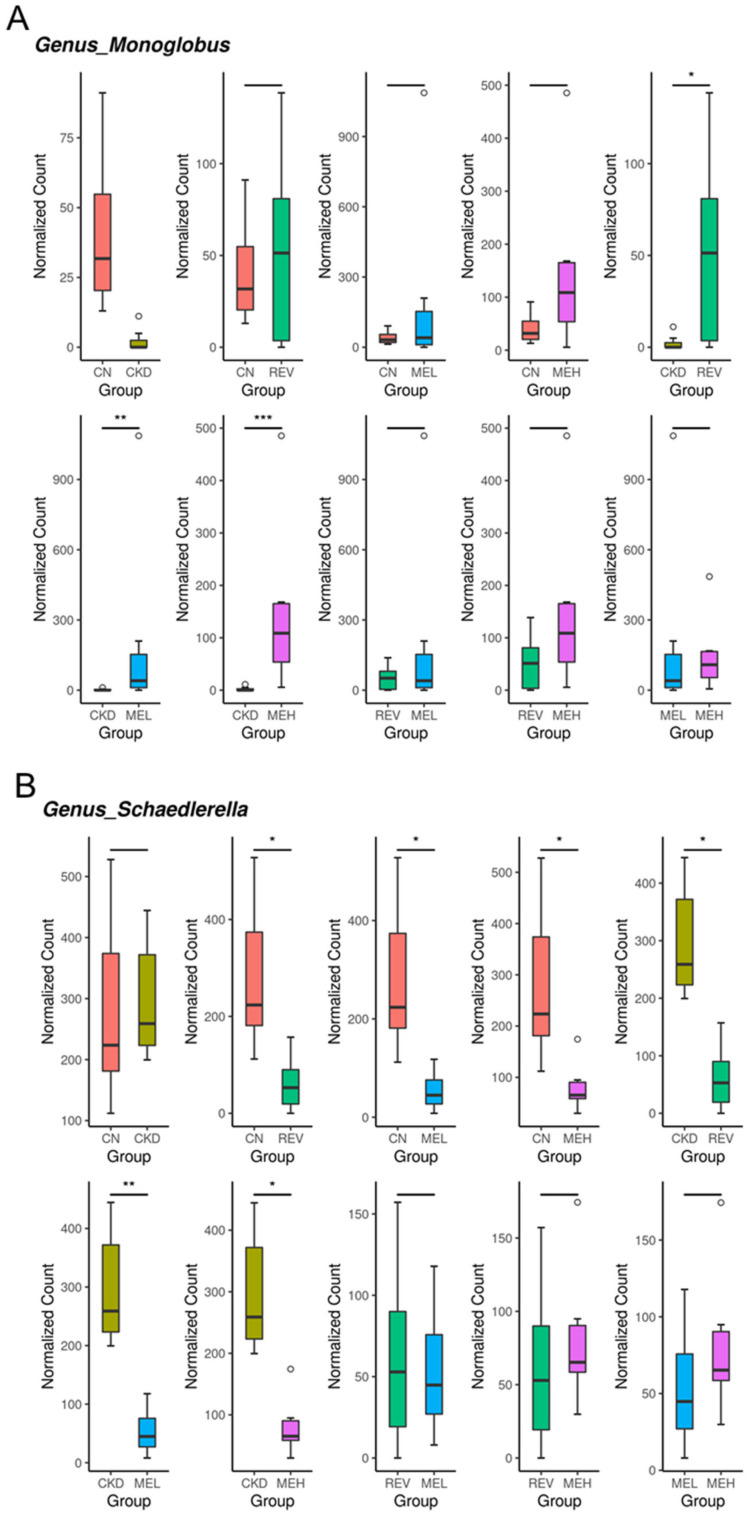
Effect of chronic kidney disease (CKD), resveratrol, and resveratrol butyrate monoester (RBM) on the gut microbiome. Relative abundance of the genera (**A**) *Monoglobus* and (**B**) *Schaedierella*. n = 8 per group; * *p* < 0.05; ** *p* < 0.01; and *** *p* < 0.001.

We further investigated the specific microbes with notable alterations in abundance at the genus level. As shown in Figure 6A, *Monoglobus* was augmented by resveratrol, MEL, or MEH treatment in CKD rats when compared with levels without treatment. Resveratrol and RBM, at both doses, similarly lowered the abundance of the genus *Schaedierella* in the controls and adenine-fed rats (Figure 6B).

## 4. Discussion

Whilst using a young rat CKD model, we found that RBM supplementation protects against hypertension and kidney dysfunction. Our key findings are specifically shown as follows: (1) adenine-fed young rats develop hypertension and kidney dysfunction, which can be prevented by dietary supplementation with RBM; (2) CKD-induced hypertension coincides with decreased NO bioavailability, the aberrant activation of the classical RAS, and alterations in gut microbiota compositions; (3) resveratrol and RBM supplementation shape distinct gut microbial compositions in CKD rats; (4) the protective effect of high-dose RBM is associated with an increased abundance of the genera *Duncaniella* and *Ligilactobacillus* and a decrease in *Eubacterium*; and (5) both resveratrol and RBM significantly increase the abundance of the genus *Monoglobus* and result in a reduction in *Schaedierella*.

Our finding supports prior research showing that adenine administration induces a state of CKD that is characterized by worsened kidney function and hypertension in both adults and children [22,23]. In the present study, adenine at 0.25% in the diet for 3 weeks triggered CKD via a ~30% decline in kidney function and hypertension. Here, we report for the first time that RBM can protect young rats with CKD against hypertension and kidney damage; indeed, the renal-protective effects of RBM were shown to be similar to those of resveratrol. A recent meta-analysis study indicated that resveratrol could possess a salutary anti-hypertensive effect, despite no beneficial effects being observed on renal function in human CKD [24]. Our findings are in agreement with the extensive body of literature that documents the health benefits of resveratrol and its derivatives in kidney disease [9,11]. We previously reported that RBE protects against adenine-induced kidney damage and hypertension [6]. This is due to the fact that RBE is a mixture of pristine resveratrol and five ester derivatives. However, it is not clear which ester derivatives exert beneficial effects. The present study extends our prior research by indicating that low-dose RBM and resveratrol produce very similar beneficial effects, with no statistically significant difference in both the BP and the kidney function being observed. Although no significant difference was found between the low- and high-dose RBM, we propose that the protective actions of RBM may arise in a dose-independent manner.

We characterized several key mechanisms participating in the development of CKD and hypertension that favor or correlate with the advantageous effects of RBM supplementation. First, our data revealed that the beneficial effects of RBM are produced together with augmented NO bioavailability. Indeed, previous research indicates that NO deficiency participates in CKD and hypertension [25,26]. Conversely, NO-based interventions could have therapeutic potential for treating CKD and hypertension [27]. Data presented in this study reinforce this notion, as high-dose RBM protects against adenine-induced CKD, which coincides with increased L-arginine concentrations and the ratio of L-arginine-to-ADMA in the plasma. Additionally, we observed that RBM has better renoprotective effects against oxidative damage, more than resveratrol was able to demonstrate (as shown by the 8-OHdG staining).

These results tied in well with our previous research showing that RBEs and resveratrol exhibit ROS-scavenging and antioxidant activities [14,28]. Nevertheless, depending on the dose and administration route, resveratrol may exhibit pro-oxidant properties, thereby leading to oxidative DNA damage [29]. Although our data suggested that low- and high-dose RBM could be associated with health benefits, the notion of whether RBM appears to result in side effects at other doses via pro-apoptotic actions deserves further evaluation. Our study proposes that oxidative stress damage is involved in CKD; however, it may not be the major protective mechanism of resveratrol in this model.

Another protective mechanism of RBM against CKD could be due to a rebalancing of the RAS axes. Our findings with regard to the RAS confirm previous reports, which showed that the aberrant activation of the classical RAS is involved in CKD and hypertension [30]. Moreover, the adenine-fed CKD rats possessed increased protein levels of PRR and AT1R, thereby indicating the activation of the classical ACE–angiotensin II (Ang II)–AT1R axis. The binding of renin to the PRR can also activate Ang II-independent pathways, thereby resulting in hypertension [31]. Conversely, the increases in PRR and AT1R were restored by RBM supplementation. These findings clearly indicate that the protective role of RBM is linked to a rebalancing of the RAS.

The protective effects of RBM are in keeping with an ever-evolving understanding of the role of resveratrol, in terms of it acting as a prebiotic for the gut microbiota. Though CKD, resveratrol, and RBM shape a distinct microbiome composition, we observed no difference in α-diversity between these groups.

A plant-based high-fiber diet appears to be beneficial for human health by promoting the development of lactic acid bacteria, such as *Ruminococcus* and *Ligilactobacillus* [32]. Our findings with regard to the effect of CKD suggests that the development of hypertension may be related to a reduction in these beneficial microbes. In contrast, high-dose RBM treatment resulted in higher quantities of the genera *Ligilactobacillus* and *Duncaniella*. As *Ligilactobacillus* has recently been gaining attention as a probiotic [33], our data raise the possibility that RBM may have prebiotic properties. Prior research suggests that *Duncaniella*, a butyrate-enriched species, contributes to colitis protection with its anti-inflammatory properties [34]. As such, additional research is required in order to illuminate whether the genus *Duncaniella* may be involved in the protective effects of RBM against CKD. According to our data, resveratrol and RBM cause increases in the genus *Monoglobus*, which is a pectin-degrading bacteria [35]. As pectin is a water-soluble fiber and is recommended for the prevention and progression of kidney disease [36], the question of whether RBM increases *Monoglobus*—thereby contributing to its renoprotective actions—deserves further clarification. Thus far, little is known about the genus *Schaedierella*. There is only a report that demonstrates that *Schaedierella* is a new genus and that it is a close relative of *Clostridium* ASF 502 [37]. Taken together, it is thus imperative to obtain a deeper understanding of their roles as microbial markers, as well as their implications in CKD pathogenesis.

Our study has certain limitations. First, the two testing doses of RBM may not be able to entirely elucidate its dose-dependent effect and thus enable one to satisfactorily compare its effectiveness with resveratrol. Additional studies with various test doses and then deciding on the ideal dose for clinical translation are warranted. Second, we restricted RBM treatment to the CKD group due to the fact that prior work has indicated that resveratrol exerts no adverse effects on normal controls [38]. Nevertheless, further investigation is still required in order to investigate the safety of RBM with respect to its consideration for future pharmaceutical use, instead of just being utilized as a dietary supplement [39]. Third, our metagenomic analysis of the gut microbiota revealed associations between the gut microbiota composition and the protective effects of RBM against CKD. Having said this, their causal relationship awaits further study. As the gut microbiota may affect host metabolic phenotypes through the production of metabolites, additional studies are required to evaluate microbial metabolites and microbial function in order to identify specific bacterial strains or metabolites that can support the causal role of the gut microbiota. Fourth, we only measured plasma creatinine levels in order to reflect renal function; a gold-standard approach would need to be more accurate in terms of measuring the glomerular filtration rate. Last, the renoprotective effects of RBM may be sex-dependent or model-dependent. Whether sex-based differences occur in the therapeutic response of RBM is a question that requires further evaluation. As such, our conclusion should be verified with a second pediatric CKD model.

## 5. Conclusions

In conclusion, dietary RBM supplementation exerts several therapeutic effects on CKD, including the restoration of NO, rebalancing of the RAS, suppression of oxidative stress, and alterations in the gut microbiota. After improving the bioavailability of resveratrol, RBM supplementation would be valuable for the purposes of optimizing kidney health and also for the extensive application of resveratrol-based natural products in clinical settings.

## Figures and Tables

**Table 1:** Primary antibodies used for Western blotting.

Protein	Host	Catalog No./Company	Dilution
eNOS	Mouse	BD610297/BD Biosciences	1:250
nNOS	Mouse	SC-5302/Santa Cruz	1:200
PRR	Rabbit	ab40790/Abcam	1:500
ACE2	Rabbit	SC-20998/Santa Cruz	1:1000
AT1R	Rabbit	AB15552/Millipore	1:500
MAS	Rabbit	SC-135063/Santa Cruz	1:1000

eNOS = endothelial nitric oxide synthase; nNOS = neuronal nitric oxide synthase; PRR = (pro)renin receptor; ACE2 = angiotensin-converting enzyme-2; AT1R = angiotensin II type 1 receptor; and MAS = angiotensin (1–7) receptor MAS receptor.

**Table 2:** Weights, BP, and renal functions of 12-week-old rats.

Groups	CN	CKD	REV	MEL	MEH
Mortality	0%	12.5%	12.5%	12.5%	12.5%
Body weight (BW), g	268 ± 12	327 ± 16 *	334 ± 7 *	374 ± 10 *	342 ± 6 *
Left kidney weight, g	1.38 ± 0.07	1.99 ± 0.1 *	1.95 ± 0.04 *	1.95 ± 0.21 *	1.9 ± 0.04 *
Left kidney weight, 100g BW	0.51 ± 0.02	0.56 ± 0.02	0.58 ± 0.01 *	0.56 ± 0.02	0.55 ± 0.01
Systolic blood pressure, mmHg	125 ± 1	143 ± 1 *	132 ± 1 #	129 ± 2 #	134 ± 1 #
Diastolic blood pressure, mmHg	83 ± 2	93 ± 3 *	81 ± 5	86 ± 2	83 ± 3 #
Mean arterial pressure, mmHg	97 ± 2	110 ± 2 *	98 ± 3 #	100 ± 2 #	100 ± 2 #
Creatinine, μM/L	16.6 ± 0.7	21.2 ± 0.7 *	17.6 ± 0.4 #	16.8 ± 0.5 #	16 ± 0.3 #

n = 8/group; * *p* < 0.05 vs. CN; and # *p* < 0.05 vs. CKD.

## Data Availability

Data are contained within the article.
